# Pleural Effusions

**Published:** 1877-01

**Authors:** 


Pleural Effusions—Their Treatment.—Dr. C. A. Ewald (*Centralblatt—Medical Reporter*) states that from a series of observations made in Frerich's wards with special reference to operative interference, he concludes:

1. In cases of serous effusion in the pleura, puncture should be performed before the third week only if life be in danger.

2. If puncture be made under exclusion of air and with previous disinfection of the instrument, no serous exudation becomes purulent.

3. The only means of determining with certainty whether a pleural effusion is serous or purulent is an exploratory puncture.

4. Incisions with puncture should be made as early as possible into purulent exudations.

5. The mortality after incision into purulent effusions is from fifty to sixty per cent. when they are treated according to the present plan (incision in the sixth intercostal space, between the nipple and the anterior axillary line, washing out with disinfectants once or twice daily, a catheter being retained in the wound, or one or more ribs resected).

6. Sanguineous effusion is always the result of malignant growths of the pleura.

7. Serous exudations do not exclude the presence of tuberculosis and cancer of the pleura.

